# The Illinois State Board of Health

**Published:** 1887-02

**Authors:** 


THE ANNUAL REPORT OF THE ILLINOIS STATE BOARD OF HEALTH, of which a brief notice appears in another part of the JOURNAL AND EXAMINER, shows the good work that is being done by that board. Whatever misgivings there were in the minds of the medical profession of this state when the board was created, they must have been dispelled long before this time, in view of the good work and constant progress made by it from year to year. Not only has the sanitary condition of the state been improved under the requirements of the board, but the status of the medical profession of the state has been elevated during that time, and not a small part of the credit for it is due that board for the work that it has done through the power vested in it under the *Medical Practice Act*. Other states have seen the beneficial results that have come from its acts, and have copied after it.

The work of compiling the names and standing of all of the medical colleges of the United States and of Canada, done by the board, is recognized as superior to anything of the kind accomplished in this country, and has called forth much praise for its thoroughness and evidence of its utility.

In the effort being made by the board to encourage more liberal education in the medical profession, and to gradually elevate the standard of requirement by medical colleges before conferring a degree which, in many cases, carries with it a right to claim a license to practice medicine in this state, and entitles the holder of it to practice without other permit in those states where other permits are not required, the board will have the approval and encouragement of the more intelligent part of the medical profession of this state and the respect of those in other states.

In view of its efficiency, its integrity, and the good work already accomplished, and still more that it is striving to accomplish, the legislature should be liberal in its appropriations for the carrying on of its work in the future.

